# Supplementary material for: Actomyosin and CSI1/POM2 cooperate to deliver cellulose synthase from Golgi to cortical microtubules in Arabidopsis
Source: Nat Commun. 2023 Nov 17;14:7442. doi: 10.1038/s41467-023-43325-9 (PMC10656550; doi:10.1038/s41467-023-43325-9)
Supplement: Supplementary file 1 — Supplementary Information [file 41467_2023_43325_MOESM1_ESM.pdf]

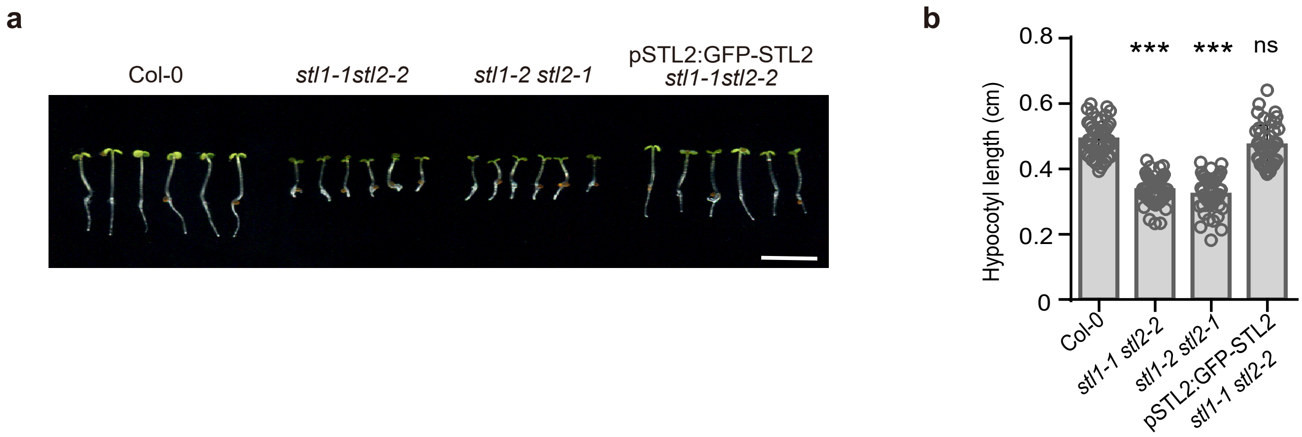


**Supplementary Fig. 1. Complementation of *stl1stl2* mutants with the *pSTL2::EGFP- STL2* construct.**
(**a**) Seven-day-old Col-0, *stl1-1stl2-2*, *stl1-2stl2-1* and pSTL2::EGFP-STL2 (*stl1-1stl2-2*) (STL2 fused N-terminally with EGFP under STL2 native promoter in the *stl1-1stl2-2* mutant background) seedlings grown on half MS medium without sucrose in short photoperiods (4 h day/20 h night). Scale bar = 0.5 cm. (**b**) Bar graph for the hypocotyl length in (**a**). Values are mean ± SD. n = 48 seedlings for Col-0; n = 58 seedlings for *stl1-1stl2-2*; n = 52 seedlings for *stl1-2stl2-1*; n = 45 seedlings for pSTL2::EGFP-STL2 (*stl1-1stl2-2*); *** P value < 0.001; ns, not significant; two-sided Student's *t*-test.

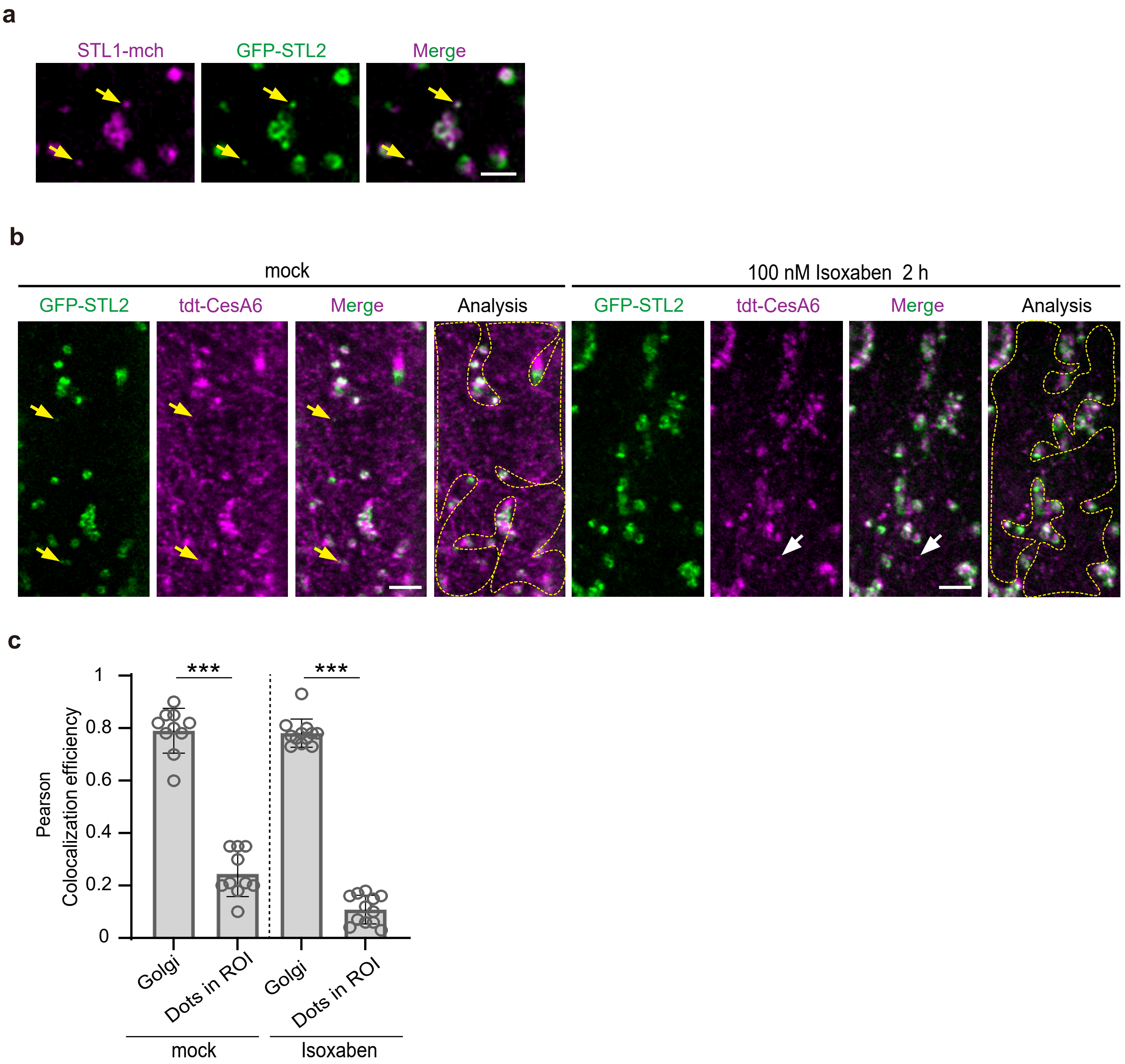


**Supplementary Fig. 2. STL1 and STL2 showed localization at Golgi and small intracellular compartments.**
(**a**) STL1-mCherry and GFP-STL2 were occasionally observed at small intracellular compartments in the epidermal cells in the top region of 4-day-old etiolated hypocotyls. Arrows indicated colocalization at the small compartments. Bar = 2 µm.
(**b**) GFP-STL2 did not show localization in isoxaben-induced SmaCCs/MASCs. Four-day-old etiolated hypocotyls were treated with 100 nM Isoxaben for 2 hours to induce SmaCCs/MASCs in epidermal cells in the top region of hypocotyls. Arrows indicate the tdtomato-CesA6 containing SmaCCs/MASCs. For analysis, region of interest (ROI) that excluded the Golgi apparatuses was marked by yellow dotted outlines. Bar = 3 µm. (**c**) Colocalization analysis of GFP-STL2 and tdTomato-CesA6 in the Golgi and ROI as indicated in (**b**) using the Pearson correlation coefficient. Values are mean ± SD. n = 10 cells from 4 mock-treated seedlings; n = 12 cells from 4 isoxaben-treated seedlings; *** P value < 0.001; two-sided Student's *t*-test.

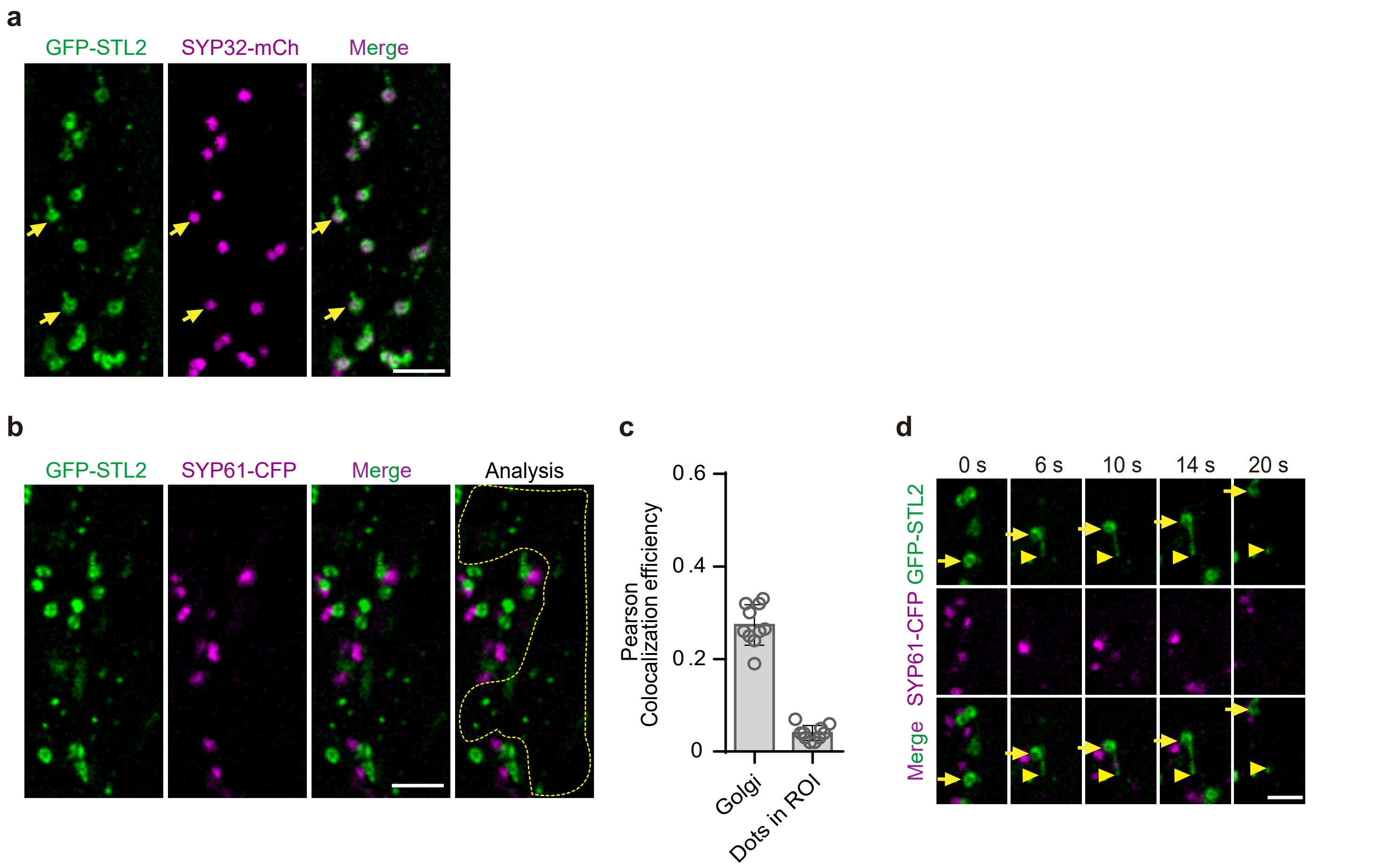


**Supplementary Fig. 3. The Golgi membrane stretching events did not involve the general Golgi marker nor the TGN.** (**a**) The Golgi membrane tail-stretching events were not observed for the cis-Golgi marker SYP32-mCherry in the basal region of 4-day-old etiolated hypocotyls. Bar = 3 μm. (**b**) The TGN marker SYP61-CFP did not colocalize with GFP-STL2 in the SmaCCs/MASCs. For analysis, region of interest (ROI) that excluded the Golgi apparatuses was marked by yellow dotted outlines. Bar = 3 μm. (**c**) Colocalization analysis of GFP-STL2 and SYP61-CFP in the Golgi and ROI as indicated in (**b**) using the Pearson correlation coefficient. Values are mean ± SD. n = 10 cells from 4 seedlings. (**d**) The TGN marker SYP61-CFP did not colocalize with GFP-STL2 in the Golgi tail during the Golgi membrane tail-stretching process. Bar = 2 μm.

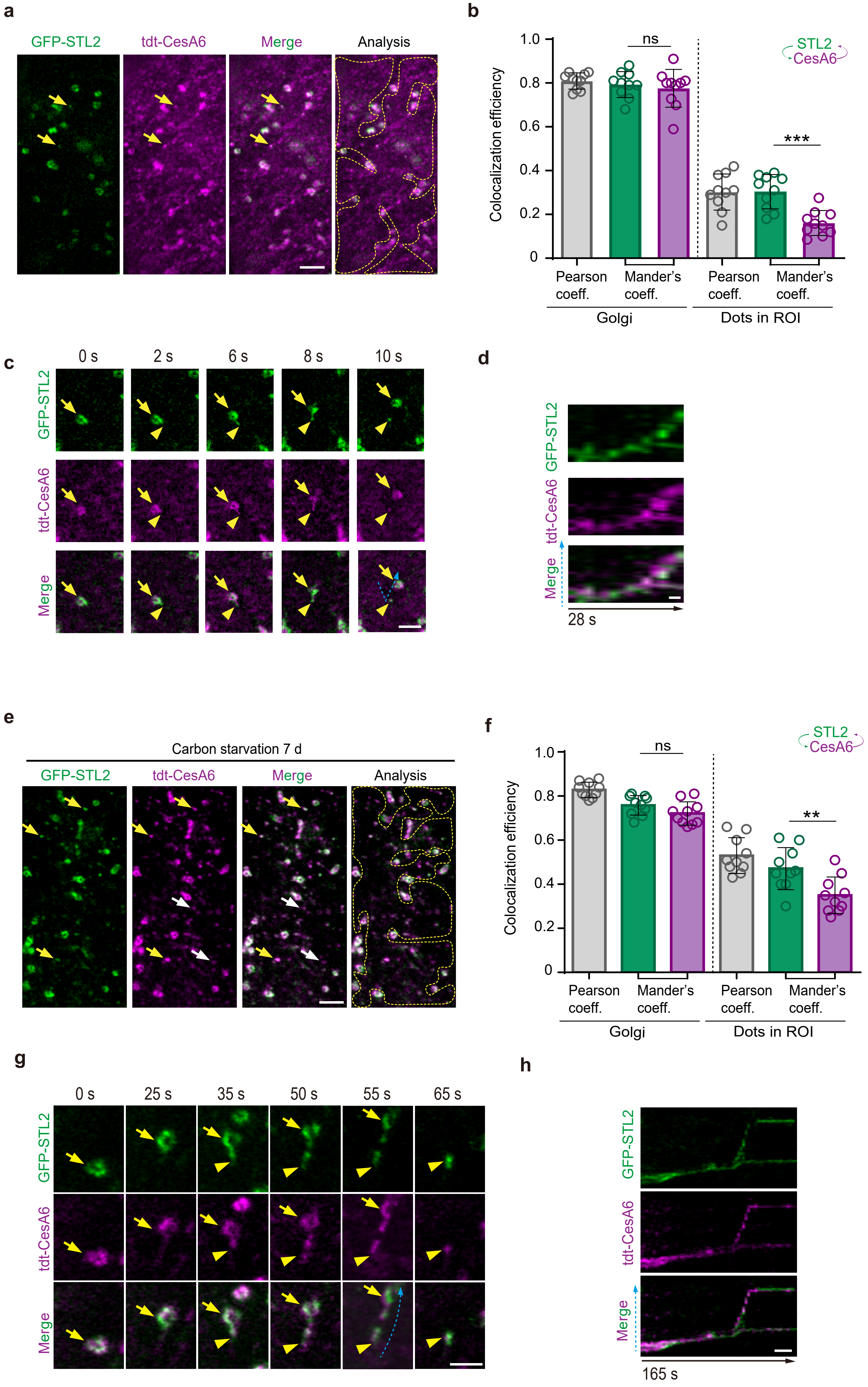
a
GFP-STL2
tdt-CesA6
Merge
Analysis
b
Colocalization efficiency
1.0
0.8
0.6
0.4
0.2
0
ns
***
STL2
CesA6
Pearson coeff.
Mander's coeff.
Pearson coeff.
Mander's coeff.
Golgi
Dots in ROI
c
0 s
2 s
6 s
8 s
10 s
GFP-STL2
tdt-CesA6
Merge
d
GFP-STL2
tdt-CesA6
Merge
28 s
e
Carbon starvation 7 d
GFP-STL2
tdt-CesA6
Merge
Analysis
f
Colocalization efficiency
1.0
0.8
0.6
0.4
0.2
0
ns
**
STL2
CesA6
Pearson coeff.
Mander's coeff.
Pearson coeff.
Mander's coeff.
Golgi
Dots in ROI
g
0 s
25 s
35 s
50 s
55 s
65 s
GFP-STL2
tdt-CesA6
Merge
h
GFP-STL2
tdt-CesA6
Merge
165 s

**Supplementary Fig. 4. STL2 localized to the SmaCCs/MASCs that were derived from Golgi in different cell types and under different growth conditions**

(**a**) Representative images showing the partial co-localization of tdTomato (tdt)-CesA6 and GFP-STL2 in the petiole epidermal cells. SmaCCs/MASCs containing both tdt-CesA6 and GFP-STL2 were indicated by yellow arrows. A region of interest (ROI) excluding the Golgi apparatuses was outlined with yellow dotted lines for analysis purposes. Bar = 3 μm.
(**b**) Colocalization analysis of GFP-STL2 and tdt-CesA6 was conducted in the Golgi and the ROI in (**a**), using the Pearson correlation coefficient and Mander's coefficient. The arrow schemes represent the overlap of GFP-STL2 intensity with tdt -CesA6 (green) and the reversals (magenta). Values are mean ± SD. n = 10 cells from 4 seedlings; *** P value < 0.001; ns, not significant; two-sided Student's *t*-test. (**c-d**) Time-lapse images (**c**) and kymograph analysis (**d**) showing that the SmaCCs/MASCs labeled with GFP-STL2 and tdt-CesA6 were derived directly from the Golgi apparatus through a membrane tail-stretching process. Bars = 2 μm. (**e**) Representative images showing partial co-localization of tdTomato (tdt)-CesA6 and GFP-STL2 in the epidermal cells in 7-day-old hypocotyls grown in short photoperiods (4 h day/20 h night) on sucrose-free media. Yellow arrows indicated the SmaCCs/MASCs containing both tdt-CesA6 and GFP-STL2, while white arrows indicated SmaCCs/MASCs without GFP-STL2. For analysis, region of interest (ROI) that excluded the Golgi apparatuses was marked by yellow dotted outlines. Bar = 3 μm. (**f**) Colocalization analysis of GFP-STL2 and tdt-CesA6 in the Golgi and ROI as indicated in (**e**), using the Pearson correlation coefficient and Mander's coefficient. The arrow schemes indicated the intensity overlap of GFP-STL2 with tdt-CesA6 (green) and the reversals (magenta). Values are mean ± SD. n = 10 cells from 4 seedlings; ** P value < 0.01; ns, not significant; two-sided Student's *t*-test. (**g-h**) Time-lapse images (**g**) and kymograph analysis (**h**) showing that the GFP-STL2 and tdt-CesA6-labelled SmaCCs/MASCs were directly derived from Golgi through a Golgi membrane tail-stretching process. Bars = 2 μm.

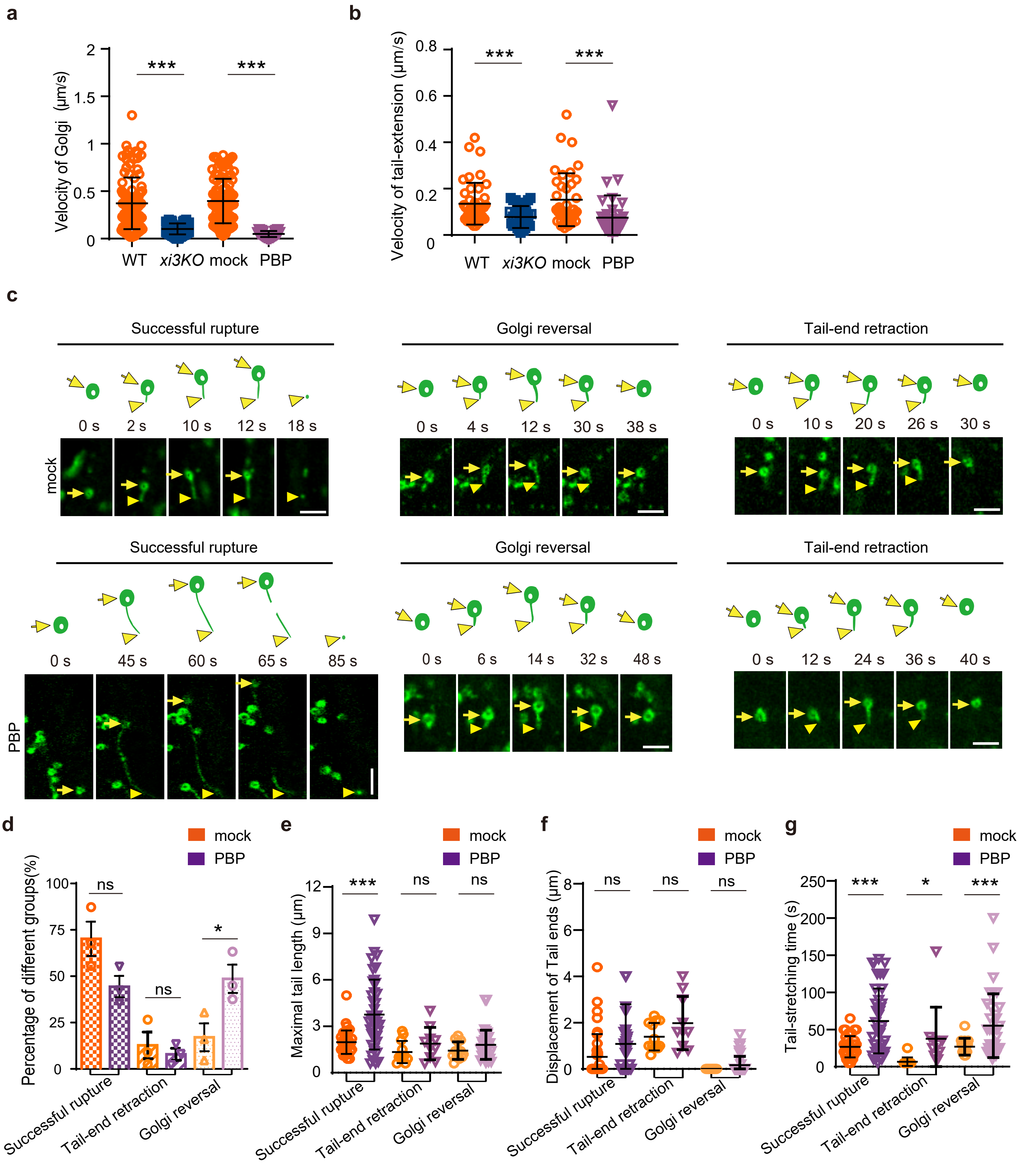


**Supplementary Fig. 5. The velocity of Golgi movement and the Golgi tail stretching processes were impaired in myosin-deficient cells.**

(**a**) Quantification of the velocity of GFP-STL2-labeled Golgi in the control, *xi3KO* mutants, mock-treated and 0.75 μM PBP -treated cells. Values are mean ± SD. n = 106 Golgi from 6 cells in 4 WT seedlings; n = 104 Golgi from 10 cells in 4 *xi3KO* seedlings; n = 106 Golgi from 8 cells in 4 mock-treated seedlings; n = 104 Golgi from 9 cells in 5 PBP-treated seedlings; *** P value < 0.001; two-sided Student's *t*-test. (**b**) Quantification of the Golgi tail-extension velocity in the control, *xi3KO* mutants, mock-treated cells, and cells treated with 0.75 μM PBP. Values are mean ± SD. n = 43 Golgi membrane tail -stretching events from 10 cells in 6 WT seedlings; n = 32 Golgi membrane tail-stretching events from 10 cells in 5 *xi3KO* seedlings; n = 39 Golgi membrane tail-stretching events from 8 cells in 3 mock-treated seedlings; n = 40 Golgi membrane tail-stretching events from 15 cells in 7 PBP-treated seedlings; *** P value < 0.001; two-sided Student's *t*-test.

(**c**) Time-lapse images showing the Golgi membrane tail-stretching processes labelled by GFP-STL2 in the mock-treated and 0.75 μM PBP-treated cells. Representative events of successful rupture, Golgi reversal and tail-end retraction were shown in the left, middle and right panels, respectively. The progress of the membrane tail-stretching events at the indicated time points was schematically presented above the images. Bars = 2 μm. (**d**) Percentage of different groups of Golgi membrane tail-stretching events in the mock-treated and PBP-treated cells. Values are mean ± SE. n = 14 cells from 6 WT seedlings examined over 3 independent experiments; n = 22 cells from 11 *xi3KO* seedlings examined over 3 independent experiments. * P value < 0.05; ns, not significant; two-sided Student's *t* -test. (**e**-**g**) Quantification of the maximal tail length (**e**), the displacement of the tail ends (**f**) and the tail stretching time (**g**) in the mock-treated and PBP-treated cells. Values are mean ± SD. In total, 64 Golgi membrane tail-stretching events in 9 mock-treated cells and 85 Golgi membrane tail-stretching events in 10 PBP-treated cells were quantified. * P value < 0.05; *** P value < 0.001; ns, not significant; two-sided Student's *t*-test.

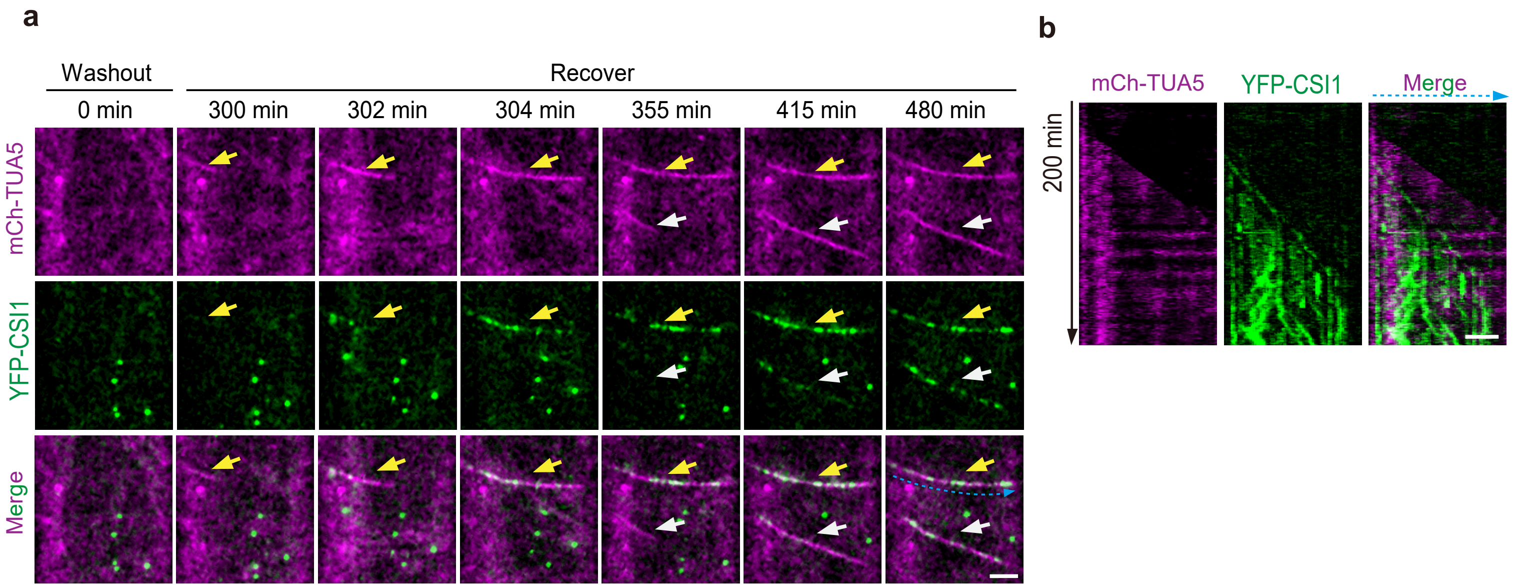


**Supplementary Fig. 6. The spatiotemporal relationship between CSI1/POM2 and cortical microtubules.**
(**a-b**) Time-lapse images (**a**) and kymograph analysis (**b**) showing that mCherry-TUA5-labeled cortical microtubules were assembled before the recruitment of 3×YFP-CSI1/POM2 to microtubules (300 min, labeled by the yellow arrow; 355 min, labeled by the white arrow). Oryzalin (20 μM) was first applied to fully depolymerize microtubules and the inhibitor was then washed out to allow the recovery of microtubules. Bars = 3 μm.

**Supplementary Table 1. Primers used in this paper.**

| Primer Name | Nucleotide Sequence | Use |
|---|---|---|
| **pSTL2_for** | GGCCAGTGCCAAGCTTCCGCAGGACACGCAGTACTTAGAGTC | Cloning of pSTL2::EGFP-STL2 |
| **pSTL2_rev** | TGCTCACCATCGGTACCCCAAATCGGAGAACACACCCCC | Cloning of pSTL2::EGFP-STL2 |
| **STL2_for** | CACCATGTTGGTTCAAGATCGTGTGGCTC | Cloning of pSTL2::EGFP-STL2 |
| **STL2_rev** | TTATACCAATTCCATCAATAGCGGATC | Cloning of pSTL2::EGFP-STL2 |
| **fABD2 _for** | GGGGACAAGTTTGTACAAAAAAGCAGGCTTCATGGATCCTCTTGAAAGAGCTGAATTGGTTCTC | Cloning of p35S:: ECFP-fABD2 |
| **fABD2_rev** | GGGGACCACTTTGTACAAGAAAGCTGGGTCTGACTCGATGGCTGCTTCC | Cloning of p35S:: ECFP-fABD2 |
| **csi1-3 _LP** | TAGGCACACCAAAAGATGAAC | Genotyping the T-DNA insertion lines |
| **csi1-3 _RP** | GAGCTACAGAGCCTGCAACAC | Genotyping the T-DNA insertion lines |
| **xi1-LP** | TCAAAACGTTGAACTAACCGG | Genotyping the T-DNA insertion lines |
| **xi1-RP** | TTGTTTGGACGGGTATCTCAG | Genotyping the T-DNA insertion lines |
| **xi2-LP** | TAGGTTTCTGGCTAGGAAGGC | Genotyping the T-DNA insertion lines |
| **xi2-RP** | CAAAGGATACCTCTGCATTGC | Genotyping the T-DNA |

| | | |
|---|---|---|
| | | insertion lines |
| **xiK-LP** | GGGTAGCAAGATACTCCTCGG | Genotyping the T-DNA insertion lines |
| **xiK-RP** | GCAAGAGCAACTCAATTCTGG | Genotyping the T-DNA insertion lines |
| **stl1-1_LP** | TTCACTTGCAAACAAAACACG | Genotyping the T-DNA insertion lines |
| **stl1-1_RP** | ACAGCAACATAACCACCAAGC | Genotyping the T-DNA insertion lines |
| **stl2-2_LP** | CAAGATCGTGTGGCTCCTAAG | Genotyping the T-DNA insertion lines |
| **stl2-2_RP** | CTCCAAGCAAGCAAGAATTTG | Genotyping the T-DNA insertion lines |
