# Supplementary material for: Actomyosin and CSI1/POM2 cooperate to deliver cellulose synthase from Golgi to cortical microtubules in Arabidopsis
Source: Nat Commun. 2023 Nov 17;14:7442. doi: 10.1038/s41467-023-43325-9 (PMC10656550; doi:10.1038/s41467-023-43325-9)
Supplement: Supplementary file 3 — Description of Additional Supplementary Files [file 41467_2023_43325_MOESM3_ESM.pdf]

Description of Additional Supplementary Files:

Supplementary Movie 1: GFP-STL2 tracked the depolymerizing ends of microtubules (corresponding to Figure 1d). Bar = 2 µm.

Supplementary Movie 2: GFP-STL2 and tdt-CesA6-labelled SmaCCs/MASCs were directly derived from Golgi through a membrane tail-stretching process (corresponding to Figure 1j). Bar = 2 µm.

Supplementary Movie 3: The Golgi membrane tail-stretching events were not observed for the cis-Golgi marker SYP32-mCherry (corresponding to Supplemental Figure 3a). Bar = 3 µm.

Supplementary Movie 4: The TGN marker SYP61-CFP did not colocalize with GFPSTL2 during the Golgi membrane tail-stretching process (corresponding to Supplemental Figure 3d). Bar = 2 µm.

Supplementary Movie 5: SmaCCs/MASCs labeled with GFP-STL2 and tdt-CesA6 were derived directly from the Golgi apparatus using a membrane tail-stretching process in petiole epidermal cells. (corresponding to Supplemental Figure 4g). Bar = 2 µm.

Supplementary Movie 6: GFP-STL2 and tdt-CesA6-labelled SmaCCs/MASCs were directly derived from Golgi through a Golgi membrane tail-stretching process under carbon limitation condition (corresponding to Supplemental Figure 4c). Bar = 2 µm.

Supplementary Movie 7: Spatiotemporal interaction among GFP-STL2-localized Golgi, CFP-fABD2-labelled actin filaments and mCherry-TUA5-labelled cortical microtubules during the Golgi membrane tail-stretching processes (corresponding to Figure 2a). The successful rupture of the membrane tail was achieved through efficient forward movement of the Golgi along actin filaments and anchor of the tail end at microtubules. Bar = 2 µm.

Supplementary Movie 8: The tail-end retraction of the Golgi membrane tail-stretching events (corresponding to Figure 2a). Detachment of the tail end from microtubules resulted in retraction of the tail back to Golgi. Bar = 2 µm.

Supplementary Movie 9: The Golgi reversal of the Golgi membrane tail-stretching events (corresponding to Figure 2a). The reversed movement of the Golgi led to backpedaling of the tail to Golgi. Bar = 2 µm.

Supplementary Movie 10: Successful rupture of the Golgi membrane tail labelled by GFP-STL2 in the xi3KO cells (corresponding to Figure 3a). Bar = 2 µm.

Supplementary Movie 11: The successful rupture of the Golgi membrane tail labelled by GFP-STL2 and mCherry-CSI1/POM2 (corresponding to Figure 4c). The successful rupture events included two subgroups: a group with mCherry-CSI1/POM2 signal existing already at the initial phase of the membrane tail-stretching processes (labelled by yellow arrows and arrowheads), and the other group without CSI1/POM2 at the tail end at the beginning of membrane tail-stretching processes (labelled by white arrows and arrowheads). Bar = 3 µm.

Supplementary Movie 12: The tail-end retraction of the Golgi membrane tail labelled by GFP-STL2 and mCherry-CSI1/POM2 (corresponding to Figure 4c). Bar = 3 µm.

Supplementary Movie 13: The tail-end retraction of the Golgi membrane tail labelled by GFP-STL2 in csi1-3 mutants (corresponding to Figure 4d). Bar = 3 µm.

Supplementary Movie 14: mCherry-TUA5-labelled cortical microtubules were assembled before the recruitment of 3×YFP-CSI1/POM2 to microtubules (corresponding to Supplemental Figure 6a). Oryzalin (20 µM) was first applied to fully depolymerize microtubules and the inhibitor was then washed out to allow the recovery of microtubules. Bar = 3 µm.

Supplementary Movie 15: Single insertion events of CSCs were observed in SmaCCs/MASCs labeled with both GFP-STL2 and tdTomato-CesA6. (corresponding to Figure 5a). Bar = 1 µm.
